# Genomic analysis of the natural history of attention-deficit/hyperactivity disorder using Neanderthal and ancient *Homo sapiens* samples

**DOI:** 10.1038/s41598-020-65322-4

**Published:** 2020-05-25

**Authors:** Paula Esteller-Cucala, Iago Maceda, Anders D. Børglum, Ditte Demontis, Stephen V. Faraone, Bru Cormand, Oscar Lao

**Affiliations:** 1 CNAG-CRG, Centre for Genomic Regulation (CRG), Barcelona Institute of Science and Technology (BIST), Barcelona, Spain; 2 Universitat Pompeu Fabra (UPF), Barcelona, Spain; 3 The Lundbeck Foundation Initiative for Integrative Psychiatric Research, iPSYCH, Aarhus, Denmark; 4 Centre for Integrative Sequencing, iSEQ, and Aarhus Genome Centre, Aarhus, Denmark; 5 Department of Biomedicine – Human Genetics, Aarhus University, Aarhus, Denmark; 6 Departments of Psychiatry and of Neuroscience and Physiology, SUNY Upstate Medical University, Syracuse, NY USA; 7 Departament de Genètica, Microbiologia i Estadística, Facultat de Biologia, Universitat de Barcelona, Barcelona, Spain; 8 Centro de Investigación Biomédica en Red de Enfermedades Raras (CIBERER), Instituto de Salud Carlos III, Madrid, Spain; 9 Institut de Biomedicina de la Universitat de Barcelona (IBUB), Barcelona, Spain; 10 Institut de Recerca Sant Joan de Déu (IR-SJD), Esplugues de Llobregat, Spain; 11 Present Address: Institut de Biologia Evolutiva (UPF-CSIC), Barcelona, Spain

**Keywords:** Evolutionary genetics, ADHD

## Abstract

Attention-deficit/hyperactivity disorder (ADHD) is an impairing neurodevelopmental condition highly prevalent in current populations. Several hypotheses have been proposed to explain this paradox, mainly in the context of the Paleolithic versus Neolithic cultural shift but especially within the framework of the *mismatch theory*. This theory elaborates on how a particular trait once favoured in an ancient environment might become maladaptive upon environmental changes. However, given the lack of genomic data available for ADHD, these theories have not been empirically tested. We took advantage of the largest GWAS meta-analysis available for this disorder consisting of over 20,000 individuals diagnosed with ADHD and 35,000 controls, to assess the evolution of ADHD-associated alleles in European populations using archaic, ancient and modern human samples. We also included Approximate Bayesian computation coupled with deep learning analyses and singleton density scores to detect human adaptation. Our analyses indicate that ADHD-associated alleles are enriched in loss of function intolerant genes, supporting the role of selective pressures in this early-onset phenotype. Furthermore, we observed that the frequency of variants associated with ADHD has steadily decreased since Paleolithic times, particularly in Paleolithic European populations compared to samples from the Neolithic Fertile Crescent. We demonstrate this trend cannot be explained by African admixture nor Neanderthal introgression, since introgressed Neanderthal alleles are enriched in ADHD risk variants. All analyses performed support the presence of long-standing selective pressures acting against ADHD-associated alleles until recent times. Overall, our results are compatible with the mismatch theory for ADHD but suggest a much older time frame for the evolution of ADHD-associated alleles compared to previous hypotheses.

## Introduction

Attention-deficit/hyperactivity disorder (ADHD) is a common neurodevelopmental condition characterised by inattention, motor hyperactivity and impulsivity^1^. Its worldwide prevalence is around 5% in children and adolescents and its symptoms often persist into adulthood^2^. Twin studies have consistently shown high heritability rates of 70–80%^3^, a considerable fraction of which is explained by single nucleotide polymorphisms (SNPs)^4^. However, it has not been until recently that genome-wide association studies (GWAS) have reported significant variants associated with ADHD^5^. In practise, ADHD manifests as a notably impairing disorder with increased risk of psychiatric comorbidity, social dysfunction, injuries from traffic accidents, medical morbidity and premature death. These impairments translate into a reduced quality of life not only to ADHD patients themselves but also to their relatives^2,6–8^.

The fact that ADHD poses such a burden on individual life quality^9^ and yet is so prevalent seems counterintuitive from an evolutionary perspective, as one might expect natural selection to purge the risk alleles from the population^10^. In this regard, several hypotheses have been put forward to explain the evolutionary persistence of this disorder, each of them emphasising on different aspects of the ADHD phenotype (Table 1). Such theories dispute that ADHD is the inevitable by-product of a continuous variable complex trait not necessarily under selective pressure^11^. Instead, they resort to the evolutionary concept of *mismatch*^12^, suggesting that traits which are specific to individuals diagnosed with ADHD increased the fitness of carriers in an ancestral environment and therefore spread in the population due to natural selection. However, as a result of the astonishingly fast cultural evolution of the human species and subsequent environmental changes, particularly in the last 10,000 years, ADHD traits have become maladaptive in present-day societies, and are therefore evolving to meet the demands of current environments^13–15^. One of these theories, the hunter-farmer theory, states that ADHD traits specific to hunter-gatherers would have been environmentally beneficial well into the Neolithic Revolution, when non-ADHD traits characteristic of agriculturalism spread. Under this view, current humans diagnosed with ADHD should be enriched in alleles from ancient hunter-gatherers (Table 1)^15^.

**Table 1:** Mismatch theories explaining the adaptive role of attention-deficit/hyperactivity disorder in ancestral environments and their expected genetic outcome in the conducted analyses.

Theory [Reference]	Prehistoric context	Description	Expected genetic outcome from ancient DNA analyses and tests on recent selective pressures
The wader theory^13^	Human speciation (> 6 mya)	Based on the *Aquatic Ape Theory*^67^, body hair loss in human females favoured them to enter water shores for protection from land predators. In this new environment, as infants could no longer cling to their mothers, ADHD traits (e.g., hypervocalisation) were able to attract maternal attention (e.g., breastfeeding, continuous monitoring) and thus more likely to survive.	1) ADHD-risk alleles are mostly the derived allele compared to chimpanzee. 2) Neanderthal-introgressed regions equally enriched in ADHD-risk and ADHD-protective alleles.
The fighter theory^13^	Coexistence with Neanderthals (~60–30 kya)	ADHD-associated aggression in ancient humans favoured Neanderthal genocide. ADHD traits of impulsiveness, reduced inhibition control and inattention are the result of a battlefield mind set.	1) Neanderthal-introgressed regions in modern humans enriched in ADHD-protective alleles. 2) Decrease of ADHD-protective alleles during the time of coexistence with Neanderthals.
The response-readiness theory^14^	Hunter-gatherers (~200–10 kya)	ADHD traits in response-ready individuals were advantageous over problem-solvers (without ADHD phenotype) in harsh and changing conditions. Main beneficial ADHD traits in the ancestral environment: (1) hyperactivity (exploratory behaviour), (2) impulsivity (ready-to-go behaviour) and (3) inattention (rapid-scanning of the surroundings)	1) Increase of ADHD-risk alleles over Pre-Neolithic times. 2) ADHD-protective alleles are recently positively selected.
The hunter-farmer theory^15^	Hunter-gatherers (most intense hunting period, ~100 kya)	Modern individuals with ADHD are considered the remains of *hunters*, opposed to non-ADHD individuals (with *farmer* characteristics). Main beneficial ADHD traits in the ancestral environment: (1) hyperactivity (energetic and tireless behaviour), (2) impulsivity (ability to change the strategy quickly) and (3) inattention (detecting changes in the environment)	1) Increase of ADHD-risk alleles over Pre-Neolithic times. 2) Hunter-gatherer genetic component correlates with the frequency of ADHD-risk alleles in humans from the Neolithic. 3) ADHD-protective alleles are recently positively selected.

Abbreviations: kya, thousand (kilo) years ago; mya, million years ago; ADHD, attention-deficit/hyperactivity disorder.

Besides natural selection, a demographic factor that could also explain the observed prevalence of ADHD in current populations is archaic introgression. The sequencing of the Neanderthal^16^ and Denisovan^17^ genomes revealed an out-of-Africa introgression of genomic segments from archaic species into the genome of anatomically modern humans (AMH)^18–22^. Moreover, even if introgressed sequences of archaic species were subjected to purifying selection shortly after admixture^18,21,23,24^, the addition of particular introgressed regions could have helped AMH adapt to this new out-of-Africa environment^25^. In fact, there is now mounting evidence of introgressed variants currently associated with human complex traits that have undergone positive selection in AMH^21,22^, including neurological and psychiatric phenotypes^22^. These results support the mismatch theory by positing that some Neanderthal alleles could have been beneficial for AMH as they moved out of Africa but became detrimental in modern environments.

Overall, mismatch-related hypotheses in ADHD have been speculative^9^, but current genomic data allows to assess the evidence supporting each proposed model. Here we study the evolutionary nature of ADHD from a genomic perspective by analysing traces of selection in AMH and assessing the role that archaic interbreeding played in the genetic variation associated with this trait. For that purpose, we used ancient samples to analyse the evolution of ADHD-associated genetic load over time, using European present-day genomes as a baseline. Moreover, we also evaluated these theories in the context of the Neolithic transition, considering African admitxure and archaic introgression.

## Results

### Evidence of selective pressures in ADHD variants and Loss of function (LoF) intolerant genes

It has been hypothesised that genetic variants associated with early-onset traits under natural selection could be enriched in genes that are intolerant to loss of function mutations^26,27^. In agreement with this hypothesis, a previous study^28^ described that genetic variants associated with schizophrenia (SCZ) are enriched in mutation-intolerant genes and in regions under strong background selection. Given that ADHD is an early-onset condition, we assessed whether a similar trend could be observed in this phenotype. MAGMA analysis^29^ statistically supported that LoF-intolerant genes (ExAC^30^ pLI ≥ 0.9) expressed in brain are enriched in variants associated with ADHD (*p* = 4.15e-3; *p* = 4.32e-3 using genes expressed in brain^28^), which is in line with previous results^5^, thus suggesting that ADHD could be under natural selection pressures. Consistently, a significant enrichment in the heritability from ADHD-associated SNPs located in conserved regions of the genome was also observed in the aforementioned study^5^.

### Contribution of ancestral variants to ADHD

In order to characterise the nature of ADHD-associated variants we analysed the contribution of ancestral alleles to the ADHD-associated alleles controlling by the minor allele frequency (MAF) of each SNP. We observed that for statistically significant GWAS SNPs at *p* ≤ 1e-8, the ancestral allele tended to be the risk allele as compared to SNPs that are not associated with ADHD in the GWAS analysis (i.e. those showing a GWAS *p* ≥ 0.9) (odds ratio, OR = 1.34; one tail *p* based on 10,000 resamples controlling by MAF < 5e-5; Supplementary Fig. S1). This suggests that ADHD-associated variants tend to be enriched for the ancestral allele.

### Time evolution of the frequency of current ADHD-risk alleles in ancient genomes

The evolution of ADHD load was assessed by computing Kendall’s τ correlation between the average frequency of currently associated ADHD alleles (at GWAS significance < 0.01) (*f*_*ADHD*_, see Methods) and date estimates of ancient European samples from three different datasets (Supplementary Fig. S2).

**Figure 1:**
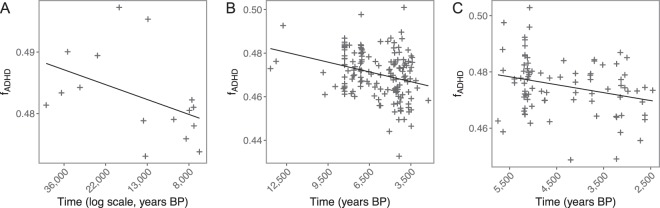
Temporal evolution of the f_ADHD_ in ancient samples using the Pre-Neolithic (**A**), Near East (**B**) and Neolithic (**C**) datasets. Each dot represents an individual defined by its *f*_*ADHD*_ and sample age. Date estimates are indicated in years BP (Before Present) and time scales are reversed to simplify interpretation. In the Neolithic dataset sample ages are shown in log scale.

Alluding to the most relevant characteristics of each dataset, we refer to these as the Pre-Neolithic^31^, Near East^32^ and Neolithic^33^ datasets. For the Near East and Neolithic datasets, which include more ancient samples, (151 and 84 genomes, respectively), we observed a significant negative τ between *f*_*ADHD*_ and sample age (Fig. 1): τ_Near East_ = −0.173 (*p* = 0.002) and τ_Neolithic_ = −0.1574 (*p* = 0.034). The Pre-Neolithic dataset (which after filtering consisted of 16 ancient samples) also showed a negative trend although it was not significant (τ_Pre-Neolithic_ = −0.317, *p* = 0.096). These results support a decrease in the frequency of ADHD alleles over time.

Since GWAS measure associations between genotypes and phenotypes in a specific environment and population sample, some studies claim that no out-of-environment nor out-of-sample extrapolation can be performed^34,35^. Hence, we inspected whether the decay trend of current ADHD-risk alleles is expected over time independently of the association of the alleles with the ADHD phenotype. For each of the three datasets, we built a Kendall’s τ null distribution assuming independence of any association between the examined alleles and ADHD by randomising the risk alleles. This approximation is equivalent to examining sets of random SNPs matched to the allele frequencies of the ADHD-associated SNPs. Rejection of the null hypothesis was reported for all three datasets (*p* of 0.046, 0.003 and 0.016 for the Pre-Neolithic, Near East and Neolithic datasets, respectively, after 10,000 randomizations) (Supplementary Fig. S3). This supports the role of ADHD in the decay of current ADHD risk alleles over time in Europe. However, ADHD shows comorbidities with other psychiatric disorders, like autism spectrum disorder (ASD) or major depressive disorder (MDD)^36^. In fact, the effect sizes of the ADHD-associated SNPs showed a relatively strong positive Kendall’s τ correlation (Kendall’s correlation of ~0.5 for all the ancient datasets) with those SNPs associated with ASD compared to other tested psychiatric disorders (Supplementary Table S1). The decay in the frequency of ADHD-risk alleles over time could be explained by demographic factors such as dilution of the ADHD-associated variants by constant admixture from a population enriched in ADHD-protective alleles. This process has been claimed as one of the reasons for the decay of Neanderthal ancestry in Europe, result of the constant migration from African populations^37^. To test this, we studied the trend of *f*_*ADHD*_ derived from European-based ADHD-associated alleles in ancient and modern African populations. Since the temporal scale of the ancient African samples and the Near East dataset overlaps, we performed a multiple linear regression of *f*_*ADHD*_ against sample age considering the continent as a covariable. This analysis showed that ancient African samples have a higher *f*_*ADHD*_ of ADHD-associated alleles than the ancient European samples (slope of the continental location variable equals 0.0238, *p* = 1.33e-12). Similarly, when we compared African and European populations in the 1000 Genomes Phase 3 dataset, African samples also showed higher *f*_*ADHD*_. This result is obtained independently of which set of SNPs defined for each of the ancient European datasets is considered (Wilcoxon test one tail *p* = 2.31e-140, one tail *p* = 1.1e-76 and one tail *p* = 8.09e-32 for Pre-Neolithic, Near East and Neolithic datasets, respectively). Additionally, the decay of ADHD-associated alleles over time could be explained by successive dilutions from European populations showing an excess of ADHD-protective alleles. Two main ancient migrations have been suggested to have shaped the genetic diversity of European populations: the steppe migration and the farmer Neolithic expansion^38^. Steppe migration cannot explain the observed sample time trend, as it considers a more recent time period than the one our samples span^38^. However, the admixture between hunter-gatherers (HG) and Neolithic farmers could explain the observed trend after the Neolithic expansion if HG had a lower burden of ADHD-associated alleles compared to Neolithic people. We observed a significant negative Kendall’s τ correlation between HG and *f*_*ADHD*_ (τ = −0.16, *p* = 0.024) in the Neolithic dataset; suggesting that Neolithic-ancestry individuals tend to carry more ADHD-risk alleles than HG. However, since the percentage of HG ancestry increases over the course of the Neolithic period^33^, sample time could act as a confounder. Therefore, we repeated the analyses controlling for sample time and no statistically significant association between both variables was observed (τ = −0.1, *p* = 0.17). Taken together, our analyses show that the observed trend between *f*_*ADHD*_ and sample time cannot be explained by ancestry dilution.

### Quantification of polygenic adaptation

**Figure 2:**
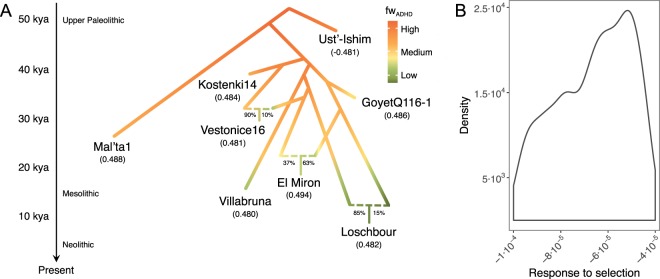
ABC-DL modelling using Pre-Neolithic samples. (**A**) Demographic model proposed by Fu *et al*. (see Figure 4 in^31^) in ancient samples from the Pre-Neolithic dataset with the mean *f*_*ADHD*_ contribution per SNP estimated using the effect size from a GWAS meta-analysis (see Fig. 1). (**B**) Posterior distribution of the parameter γ that quantifies the deviation of the frequency of the ADHD risk allele at locus *j* per generation due to selective pressures.

The previous correlation analyses between *f*_*ADHD*_ and sample age did not consider that the ancient populations might be related over time. We estimated the mean increase in allelic frequency of ADHD alleles per generation including the relationship between ancient populations in the Pre-Neolithic dataset in an Approximate Bayesian Computation coupled to Deep Learning (ABC-DL) using a simple model that considers the average selective pressure acting over all the loci and genetic drift (Fig. 2). After implementing the ABC-DL approach (see Methods), we first computed the *factor 2* statistic on 1,000 simulations to quantify the performance of the framework for properly estimating the slope. This statistic quantifies the number of times that the estimated mean from the posterior distribution is within the range 50% and 200% of the true value of the parameter used for the simulation^39^. The mean of the posterior distribution computed by ABC-DL falls within the *factor 2* statistic 93.1% of the times, thus suggesting high confidence in using the mean of the posterior distribution as a proxy of the real value.

When applied to the observed data, the posterior distribution of the trend of the allelic frequency of each ADHD-risk allele per SNP and generation had a mean of −7.72e-5 (95% Credible Interval ranging from −9.87e-5 to −5.9e-5). This result corresponds to an average decay of 0.12 in the frequency of each ADHD-increasing risk allele since Palaeolithic times until present (~1,552 generations assuming a generation time of 29 years^40^) and supports the existence of a selective pressure towards current protective ADHD alleles from Paleolithic to Neolithic times.

To further evaluate whether selective forces acted on ADHD-associated alleles for the last 2,000–3,000 years, we used the trait Singleton Density Score (tSDS) computed on the UK10K data matched by the ADHD-increasing risk allele^41^. tSDS is suited for detecting polygenic adaptation acting less than 100 generations ago, and it is robust against putative confounder effects due to background selection^41^, which is expected to act on conserved genomic regions. Given that most complex-trait heritability is caused by SNPs that do not reach GWAS significance^5,42,43^, we tested for genome-wide rank correlation between tSDS and GWAS p-values considering all SNPs^44^. The Spearman correlation observed between tSDS and GWAS p-values was not expected under neutrality (Spearman ρ = 0.197, *p* = 0.0217). This positive correlation indicates that statistically significant p-values tend to be enriched by negative tSDS values, and non-significant p-values by tSDS close to 0. Positive values of tSDS imply that the frequency of the ADHD-risk allele increased in the past, whereas negative tSDS imply that the ADHD-protective allele is increasing in frequency^44^. Our result supports that polygenic adaptation recently shaped the frequency of alleles that are protective for ADHD in one modern European population.

### Current ADHD risk alleles in the context of archaic species

Genes involved in behaviour show differences between Neanderthal and modern humans^45^ and current ADHD prevalence could potentially explain Neanderthal genocide (see the fighter theory in Table 1). To assess the role of archaic introgression in the loading of ADHD alleles into the *Homo sapiens* genome we estimated the mean effect size ($\bar{{\rm{\beta }}}$) of variants reported to be of Neanderthal origin. We used 1,151 tag SNPs from the Neanderthal introgressed haplotypes. We observed an increased average effect size compared to a null distribution estimated considering that Neanderthal introgressed alleles and ADHD effect are independent ($\bar{{\rm{\beta }}}$ = 0.013, *p* < 5e-5, Supplementary Fig. S4). This suggests that variants of Neanderthal origin are enriched in ADHD-risk alleles.

## Discussion

Our analyses support that natural selection has been acting against current ADHD-risk alleles for a long time, albeit the fraction of the causality explained by these SNPs remains relatively modest^5^ and the considered SNPs are most likely just linked to the actual causal variants.

First, we show that LoF-intolerant genes are enriched in signals of association with ADHD; given that ADHD is an early-onset condition, such result would support the hypothesis that it is under selective pressures^26,27^. Second, the ancestral state of current ADHD-associated variants is enriched in risk alleles compared to the expected under neutrality. Finally, the relationship between sample age and *f*_*ADHD*_ computed in ancient samples consistently points towards a higher frequency of ADHD-associated alleles in older samples and a negative trend with decreasing *f*_*ADHD*_ in time in these samples (Fig. 1). We showed this decay cannot be explained by dilution due to admixture with hunter-gatherers or by continuous migration from African populations carrying lower levels of the considered ADHD-risk alleles, as African populations show higher frequencies of these alleles compared to European populations. These results do not imply that African populations have a higher prevalence of ADHD, as these markers have been identified in European-ancestry samples and it has been recently shown that GWAS-SNP portability among continents is limited^34,35^. Moreover, by means of ABC-DL, we directly evidenced polygenic selective pressures against ADHD-risk alleles from 45,000 to 10,000 years. In fact, this pattern could also be traced back up to 2,000–3,000 years by using the tSDS estimates in a specific modern European population from the UK. We also proved that the decay of ADHD-risk alleles is not the demographic by-product of Neanderthal introgression, as we observed that introgressed Neanderthal alleles at SNPs that have been associated with ADHD are enriched for the risk allele instead. Once again, these results do not imply that ancient AMH or archaic species had an increased risk for ADHD, since the specific loci associated with the ADHD-like phenotype in the Neanderthal and ancient samples are unknown. However, we can make use of these results to contextualise the evolution of current ADHD variants in AMH.

Out of all the proposed hypotheses to explain the high prevalence of ADHD in current populations (Table 1), the fighter hypothesis, which assumes that ADHD-risk alleles are of anatomically modern human origin, does not predict the observed excess of Neanderthal introgressed ADHD alleles, nor the decay of *f*_*ADHD*_ in the period in which Neanderthals and AMH shared environment. The wader theory, which explains the rise in frequency of ADHD in the *Homo* lineage living in aquatic environments, cannot account for the enrichment of derived alleles associated with the protective ADHD phenotype compared to chimpanzees, nor for the differences in the proportions of introgressed ADHD-risk and ADHD-protective alleles either. This is because both Neanderthals and AMH share a common recent ancestor with respect to chimpanzees, yet with these analyses we see genomic differences in their ADHD load. Moreover, the response-readiness theory, which links ADHD to the hunter-gatherer environment, fails to explain why *f*_*ADHD*_ decreases over time during Pre-Neolithic times.

Our results on the decay of *f*_*ADHD*_ over time and the identification of recent signatures of positive selection are compatible with the mismatch hypothesis. Namely, the frequency of current ADHD-risk alleles was higher in the past, but these alleles became maladaptive due to recent environmental changes, to which Western European populations are still adapting. Within this context, the hunter-farmer hypothesis predicts a recent shift in the selective pressures due to agriculture during the Neolithic. However, this hypothesis cannot explain the observed negative association between the amount of hunter-gatherer ancestry and *f*_*ADHD*_ alleles in Neolithic samples. In fact, this result would be compatible with the hypothesis that selective pressures against current ADHD risk alleles were stronger in Paleolithic hunter-gatherers from Europe compared to Neolithic farmers from the Fertile Crescent. Nevertheless, the fact that the correlation is non-significant after controlling by sample time suggests that both populations were subjected to the same selective pressures. Furthermore, the hunter-farmer hypothesis cannot explain why current ADHD-risk alleles would have not been beneficial at least for the past 45,000 years, as this is the estimated age of the oldest sample included in our analyses. If selective pressures are acting on ADHD, our results call into question evolutionary hypotheses claiming the Neolithic Revolution to be the turning point for ADHD pressures. This conclusion falls in line with a study that suggested, after computing genetic risk scores on ancient human samples, a general improvement of the genomic health of ancient individuals over time. However, this trend was not seen for specific disease types such as cardiovascular disorders or neurological/psychological ones^46^.

The exact biological and environmental factors that fuelled the selective pressures over current ADHD-risk alleles through time remain to be elucidated. ADHD is known to be comorbid with many other psychiatric phenotypes such as major depressive disorder or ADS^47^. Notably, we observed a considerable correlation of the effect sizes of ADHD with ASD. Furthermore, within the ADHD phenotype several characteristics are plausible candidates for claiming selective pressures in AMH^14^. Moreover, ADHD shows significant genetic risk correlations with phenotypes that are strong candidates for being under selective pressure (both negative and positive) such as weight-related traits, reproductive traits and parental longevity traits^5^.

Increasing knowledge on the biology of the disease and availability of high-quality ancient genomic data will pave the way to new studies on the evolutionary factors shaping the variation of ADHD-risk alleles, whose genetic history has been depicted for the first time in this study.

## Methods

### Human genome databases

#### Ancient AMH genomes

Genotype data from 51^31^, 281^32^ and 180^33^ European ancient AMH were included in the analyses (Supplementary Table S2). Alluding to the most relevant characteristics of each dataset, we refer to these as the Pre-Neolithic^31^, Near East^32^ and Neolithic^33^ datasets. Altogether, these three datasets include *Homo sapiens* samples spanning a time period from approximately 45,000 to 2,500 years ago (Supplementary Fig. S2).

Due to low-quality DNA, for most of the ancient samples only one DNA strand could be recovered and genotyped, resulting in hemizygous-like genomes. Additionally, given the heterogeneity of procedures for data production, each dataset was analysed independently. For the Near East dataset, three individuals (Ust’-Ishim, MA1, Kostenki14) were withdrawn from analyses as their sample dates were considered to strongly deviate from the remaining individuals in the dataset.

The number of tagSNPs obtained after the linkage disequilibrium (LD) pruning present in our ancient sequences was enriched by implementing a two-step filtering method. First, individuals with a number of genotype missing genotypes above 50% were removed. After such filtering, 16, 151 and 84 ancient genomes remained in the Pre-Neolithic, Near East and Neolithic datasets, respectively. Then, variants with genotype information below 90% of the remaining individuals were discarded. The resulting variants then underwent LD pruning using PLINK 1.9^48^ with parameters *-indep 50 5 2*. ADHD-associated variants with an effect size p-value below 0.01 were retained (169,541 variants) and once filtered by LD, 3,276, 2,707 and 3,320 SNPs were obtained for the Pre-Neolithic, Near East and Neolithic datasets, respectively. The divergence in the number of SNPs is explained by the difference in the individuals included in the datasets.

Genotypes from 16 ancient African samples were retrieved^49^: 6 east Africans (Tanzania, Kenya and Ethiopia), 7 south-central Africans (Malawi), and 3 southern Africans (South Africa). Prehistoric African sample dates range from ~8,100 to 400 years ago.

#### Modern AMH genomes

Modern human genomes of European and African origin were retrieved from 1000 Genomes Phase 3^50^ (Supplementary Table S2). The database we used comprised information of 503 individuals from five European populations (CEU, Utah residents with Northern and Western European ancestry; TSI, Toscani in Italia; FIN, Finnish in Finland; GBR, British in England and Scotland; and IBS, Iberian population in Spain) and 661 individuals from 7 African populations (YRI, Yoruba in Nigeria; LWK, Luhya in Kenya; GWD, Gambian in Gambia; MSL, Mende in Sierra Leone; ESN, Esan in Nigeria; ASW, Americans of African Ancestry in SW USA; and ACB, African Caribbean in Barbados).

### SNPs datasets

#### Loci associated with ADHD and other psychiatric disorders

The effect sizes (log(Odds Ratio) or β) of ADHD association estimated in 8,047,421 SNPs were retrieved from a GWAS meta-analysis that included 11 cohort samples (19,099 cases and 34,194 controls) of European ancestry^5^ (Supplementary Table S2). Further analyses with this dataset included ADHD-associated SNP variants with a p-value of the association’s effect size below 0.01, unless specified otherwise. The GWAS results for autism spectrum disorder (ASD)^51^, bipolar disorder (BD)^52^, major depressive disorder (MDD)^53^ and schizophrenia (SCZ)^54^ were retrieved from the Psychiatric Genomics Consortium (https://www.med.unc.edu/pgc/).

#### SDS from UK10K data

A list of trait singleton density score (tSDS) values calculated using samples from the United Kingdom^41,44^ was available through the Dryad Digital Repository^44^ (Supplementary Table S2). Singletons were restricted to single nucleotide variants (i.e., excluding indels) thus resulting in a final dataset of 4,451,435 SNPs with valid SDS calls and a mean singleton distance of 1.4 Mb^44^.

#### Dataset of Neanderthal-introgressed tagSNPs in modern humans

TagSNPs of Neanderthal-introgressed haplotypes present in current human populations were retrieved from^23^ (Supplementary Table S2). Such variants were found after sequencing 1,523 geographically diverse non-African individuals. In aggregate, 1,340 Mb of the Neanderthal genome were recovered at a false discovery rate (FDR) of 5%^23,55^.

### Analyses

#### LoF-intolerant gene enrichment of ADHD-risk variants

In order to test whether ADHD-associated variants were enriched in LoF-intolerant genes, we used the list of genes defined as LoF (pLI > 0.9) from ExAC^30^ (downloaded from https://gnomad.broadinstitute.org/downloads) and conducted a MAGMA analysis^29^; we repeated the MAGMA analysis only using genes expressed in brain (i.e. the threshold to set if a gene was expressed in a given tissue is ≥ 1 FPKM).

#### Calculation of the mean frequency of ADHD-risk alleles (*f*_*ADHD*_)

For each ancient sample, a mean frequency of the number of ADHD-risk alleles (*f*_*ADHD*_) was computed by combining information from genotype and p-value. This *f*_*ADHD*_ was calculated for each individual (*i*) as the average of the set of ADHD-associated SNPs (*p* < 0.01) with genotype call in that individual (*G*_*i*_) according to:

1 $${f}_{ADHD,i}=\frac{1}{{J}_{i}}\mathop{\sum }\limits_{{\rm{j}}=1}^{{J}_{i}}{G}_{{\rm{i}},{\rm{j}}}$$

where *G*_*i,j*_ corresponds to the number of copies of the risk allele at locus *j* in individual *i* (0, 1 or 2).

#### Contribution of ancestral alleles to ADHD

In order to define the ancestral allele of the SNPs used in^5^ we used the allele present in the chimpanzee (*Pan Troglodytes*) as a proxy. To obtain the chimpanzee allele we used the alignment between panTro4 and hg19 that is publicly available through the UCSC (http://hgdownload.cse.ucsc.edu/goldenPath/hg19/vsPanTro4/reciprocalBest/). We downloaded the alignments corresponding to the 22 autosomes present in humans in AXT format. Using a custom Python script, we obtained a FASTA file with the genome of the chimpanzee (panTro4) in the human genome coordinates (hg19). Then we created a BED file that contained the positions of the SNPs and the corresponding chimpanzee allele. We merged this file with the VCF that contains the SNPs being considered, adding the ancestral allele as an extra individual in the last position. In the cases where the result of the alignment was not conclusive, the genotype was labelled as *missing*. Moreover, if information of ancestral allele was not available, the SNP was not considered.

In order to quantify whether ADHD-risk alleles tend to be enriched for the derived or ancestral state, we inferred the ancestral state of 519,911 SNPs that do not contain A/T or C/G alleles and with a GWAS *p* ≤ 1e-8 or with a GWAS *p* ≥ 0.9, suggestive of strong GWAS association with ADHD or with no association, respectively. For each ADHD GWAS-association category, we computed the percentage of SNPs showing the ancestral state as the GWAS-risk ADHD allele, and calculated the odds ratio (OR). Since the power for detecting an association depends on the minor allele frequency (MAF) of each SNP, in order to estimate how likely is to observe such OR by chance controlling for MAF, we classified each SNP by MAF in bins of 0.02. Statistical significance was obtained out of 10,000 resamples. For each resample, we randomly distributed the SNPs of each MAF bin in the two GWAS-statistical significance categories while keeping the proportion of SNPs at each category. We then computed the OR with the resampled dataset and compared with the observed OR.

#### Evaluation of *f*_*ADHD*_ trend with time in ancient samples

In order to assess the relationship between the computed *f*_*ADHD*_ and sampling age, we computed the Kendall’s τ correlation with the date estimate of the considered samples. We considered Kendall’s τ correlation for these analyses as it allows for a more direct interpretation of the estimated value^56^. To test whether the estimated correlation between sample age and *f*_*ADHD*_ is expected under the null hypothesis of independence of the risk allele and the ADHD phenotype (that is, the observed trend is due to demographic factors), for each SNP we randomly assigned the ADHD increasing risk allele, computed a new *f*_*ADHD*_ for each ancient individual and estimated the Kendall’s correlation between the new *f*_*ADHD*_ and the age of the samples. This process was repeated 10,000 times and an empirical p-value was obtained out of this null distribution as the number of times the simulated correlation was greater than the observed one. In the case of the Near East dataset, an additional analysis was conducted to test whether the observed trend could be explained by constant migration from African populations with a putatively lower *f*_*ADHD*_ than European populations. Taking advantage of the ancient African dataset, which covers similar sampling ages to the one from Near East, we computed a multiple linear regression between *f*_*ADHD*_, the continent of origin.

#### Evaluation of *f*_*ADHD*_ in modern African and European samples

We further computed the *f*_*ADHD*_ in each African and European samples from the 1000 Genomes Phase 3 using the SNPs from each ancient dataset. The difference between *f*_*ADHD*_ was tested by means of a Wilcoxon test as implemented in R.

#### Relationship between Hunter-gatherer ancestry and ADHD in ancient genomes

Aiming to validate some of the mismatch theories (Supplementary Table S2) which mainly ascribe ADHD-specific traits to foraging societies^15^, Kendall’s correlation was computed between hunter-gatherer ancestry proportions (HG) reported for the Neolithic ancient genomes^33^ and *f*_*ADHD*_. Since both variables covariate with sampling age, we also estimated the partial Kendall’s correlation between HG and *f*_*ADHD*_ controlling by sampling age using the *pcor.test *from the *ppcor* package in R.

#### Correlation between the effect sizes of ADHD and other psychiatric disorders

The Kendall’s *τ* correlation between the effect sizes of each disorder compared with ADHD was computed for the SNPs available in the Pre-Neolithic, Neolithic and Near East datasets weighted by the –log(P value) of the ADHD-associated SNP using the *test_indep* function from the *wdm* package in R.

#### Approximate Bayesian Computation coupled to a deep learning (ABC-DL) framework for estimating the ADHD selection coefficient

In order to estimate the temporal trend of the mean *f*_*ADHD*_ over all loci in ancient samples taking into account the fact that the considered populations are related, we implemented an ABC-DL framework. ABC is a statistical framework for computing the posterior distributions of parameters/models when the likelihood of the data given the parameters is not known but there is a way of generating simulated data using parameter values from prior distributions^57^. Here, we constructed a frequency-based simulator using the framework proposed by Pickrell and Pritchard^58^ to explain the change in allelic frequencies under neutrality, we define the evolution of the frequency of the ADHD-risk allele at each locus j (${f}_{ADHD,j}^{t\text{'}}$) at *t’* generations given that it was known *t* generations ago as following a Brownian movement:

2 $${f}_{ADHD,j}^{t{\prime} }\sim N({f}_{ADHD,j}^{t{\prime} +\varDelta t}+\gamma \varDelta t,\,d\varDelta t)$$

$$\varDelta t=t-t{\prime} $$

where $\gamma$ is a parameter quantifying the deviation of the frequency of the ADHD risk allele at locus *j* per generation due to selective pressures and *d* is a parameter quantifying the amount of drift between time *t* and *t’*. Values of $\gamma$ > 0 indicate positive selection for the risk ADHD allele, whereas negative values indicate positive selection for the protective ADHD allele. A value of $\gamma =0$ indicates that the frequency of the considered locus evolves under neutrality. Given a known population tree topology, Eq. (2) provides an efficient way to simulate the ${f}_{ADHD,i,j}^{t}$ at each locus and sample at time *t*. The average ${f}_{ADHD,i}^{t}=\frac{1}{{J}_{i}}\mathop{\sum }\limits_{{\rm{j}}=1}^{{J}_{i}}{f}_{ADHD,i,j}^{t}$ for each individual *i* is computed assuming that each locus contributes equally to the phenotype, since we are interested in the average temporal trend, not in the specific value of each locus. We use the tree topology for the samples from Fu *et al*. (as depicted in Figure 4 in^31^) for samples *UstIshim*, *Malta1*, *Kostenski14*, *GoyetQ116-1*, *Villabruna*, *Loschbour*, *Vestonice16* and *ElMiron* in our simulations. We used Eq. (2) to generate simulations sampling the parameters from the prior distributions described in Supplementary Table S3; from all the considered parameters, we are only interested in $\gamma$. For this parameter, we set a uniform prior distribution ranging between γ = −1e-4 and γ =1e-4 per generation, corresponding to an average change in frequency of each ADHD-risk allele between −0.15 to 0.15 in ~1,552 generations.

A key point of ABC is the definition of a proper set of non-redundant SS that recapitulates the parameters of interest. However, ascertaining informative non-redundant SS is traditionally difficult. Recently, Bai *et al*.^59^ proposed coupling ABC to Deep Learning (DL) in order to ascertain the most informative summary statistics out of the data. A DL or a set of DLs are trained with a raw representation of the data (raw-SS) to predict as an output the parameter that produced the simulation. During the training phase, raw-SS is non-linearly transformed by the DL to predict the value of the parameter used for the simulation. In a further replication step, new simulations are generated and the DL is used to predict the value of the parameter. This predicted value is then used as summary statistic (SS-DL) in the classical ABC framework. This methodology has been recently applied in population genetics to distinguish among competing demographic models and estimating demographic parameters using whole-genome data^60,61^. The ${f}_{ADHD,i}^{t}$ estimated at each ancient sample was used as the raw-SS input of the DL after standardizing each variable. In order to generate a consensus prediction of $\gamma$, we trained 10 independent DL using 10,000 simulations. Each DL was constructed in Encog 3 software^62^ using a feedforward architecture of four hidden layers of five neurons each layer. Neurons from the hidden layers used Elliott activation function^63^ with a slope of 0.1 to prevent signal saturation. The DL was trained using resilient propagation with RPROP- and a Dropout rate of 0.1^64^. Each DL was trained until the error was ≤0.01 or reached 10 000 iterations. The output was from an Elliott activation function and consisted on the value of *γ* used for the simulation scaled to range between 0 and 1. We used this prediction as SS in the ABC framework. ABC was conducted using the *abc* R package^65^ with 1,000,000 new simulations, retaining the 1,000 closest and applying the local linear regression algorithm^66^.

#### Signatures of recent (2,000–3,000 years) polygenic adaptation

To identify evidence of recent polygenic adaptation in ADHD during the last 2,000–3,000 years, the SDS statistic estimated for the UK10K data was used. Note this time-span does not correspond to a time-limited phenomenon but to the upper-limit resolution of the applied test^44^. Following the approach in^44^, trait-SDS (tSDS) estimates were obtained by matching the SDS value of the ADHD-risk allele. We clustered the tSDS values in bins of 1,000 SNPs according to their GWAS p-value. We computed the Spearman rank correlation between the tSDS values and the p-value of the association with ADHD. In order to assess whether the estimated Spearman rank correlation was expected under the null hypothesis of neutrality, we generated the null distribution out of 1,000 randomisations for each of which we split the genome in bins of 1 Mb, switched the risk allele of the SNPs of each bin with probability 0.5 and computed a new Spearman rank correlation. The resulting distribution was approached to a normal distribution and a one-tail p-value was computed.

#### Contribution of Neanderthal haplotypes to current ADHD-associated genomic diversity

A list of reported tagSNPs of Neanderthal introgressed haplotypes in modern humans^23^ (Supplementary Table S2) was used to test for an enrichment of Neanderthal variants among ADHD risk alleles. Of these tagSNPs, only those variants that were described as homozygous in the Neanderthal Altai genome were retained and the effect size of the association at introgressed Neanderthal alleles were retrieved from the ADHD GWAS meta-analysis dataset. Aiming to inspect whether the Neanderthal-introgressed haplotypes had in aggregate a real effect on ADHD (either by being more disease-associated or protective variants than expected under the hypothesis of no association with the phenotype), the allele effect size of each of the previously analysed tagSNPs was permuted at random, and the mean β of the permuted SNPs was estimated. This process was repeated 10,000 times and simulated mean β values were used to build a null distribution.

## Supplementary information

Supplementary Information.
