# Supplementary material for: Genomic analysis of the natural history of attention-deficit/hyperactivity disorder using Neanderthal and ancient Homo sapiens samples
Source: Sci Rep. 2020 May 25;10:8622. doi: 10.1038/s41598-020-65322-4 (PMC7248073; doi:10.1038/s41598-020-65322-4)
Supplement: Supplementary file 1 — Supplementary Information. [file 41598_2020_65322_MOESM1_ESM.pdf]

Supplementary Information for

Genomic analysis of the natural history of attention-deficit/hyperactivity disorder using Neanderthal and ancient *Homo sapiens* samples

Paula Esteller-Cucala, Iago Maceda, Anders D. Børglum, Ditte Demontis, Stephen V. Faraone, Bru Cormand*☯, Oscar Lao *☯

☯These authors contributed equally to this work

*Correspondence: bcormand@ub.edu (B.C.); oscar.lao@cnag.crg.eu (O.L.)

This PDF file includes:

Supplementary Figures S1 to S4

Supplementary Tables S1 to S3

Supplementary Figure S1. Null distribution of odds ratio between the percentage of ancestral alleles that are ADHD-risk alleles for SNPs that do not contain A/T or C/G alleles and with a GWAS p-value ≤ 1e-8 and with a GWAS p-value ≥ 0.9 controlling for MAF after 10,000 permutations.

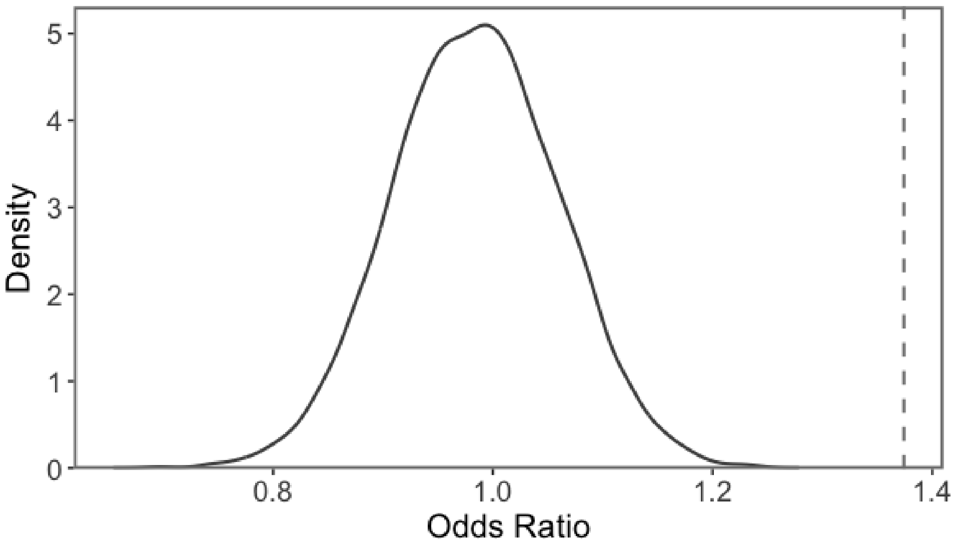

Supplementary Figure S2. Geographic location and estimated age of West Eurasian humans. Geographic distribution and age of samples from (A) the Pre-Neolithic dataset (16 samples), (B) Near East dataset (151 samples) and (C) Neolithic dataset (84 samples) considered for analyses. Each coloured dot corresponds to an age-defined individual (years BP, before present). Geographical locations were jittered to simplify the interpretation of densely sampled locations.

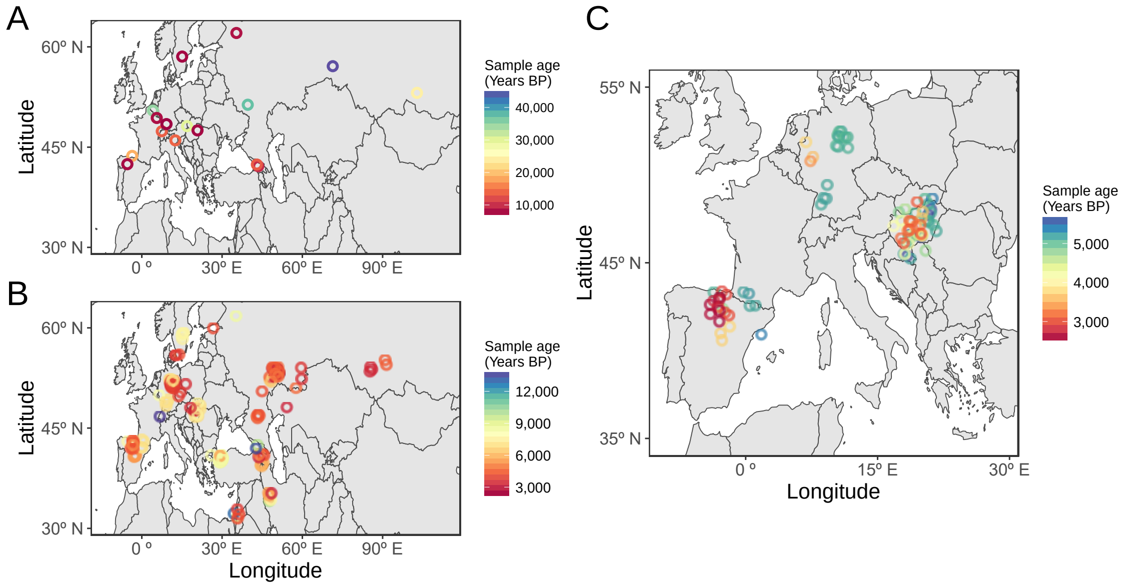

Supplementary Figure S3. Null distributions of the Kendall τ correlation coefficients between sample age and f_{ADHD} generated after 10,000 permutations for (A) the Pre-Neolithic, (B) Near East and (C) Neolithic dataset.

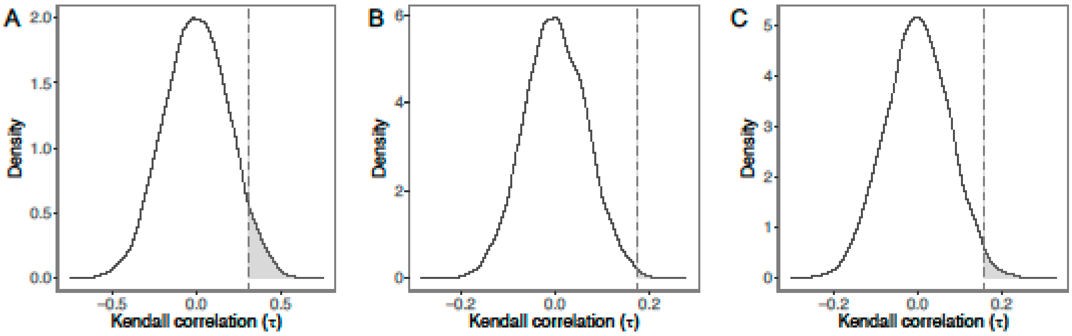

Supplementary Figure S4. Null distribution of mean β generated from 10,000 permutations of the introgressed tagSNPs. Given that the subset of considered variants had a p-value < 0.01, effect sizes close to zero (i.e., with an odds ratio of approximately 1) were not present in the distribution. To overcome the discontinuity of the distribution, all β values were transformed to fit a zero-centred distribution from which the mean β for the observed data was computed. For each round of permutations, a simulated dataset was obtained by randomly assigning the effect size of each allele from each of the 1,151 considered tagSNPs and the mean β of the resulting dataset was then calculated. The dashed line represents the mean β observed in the actual dataset.

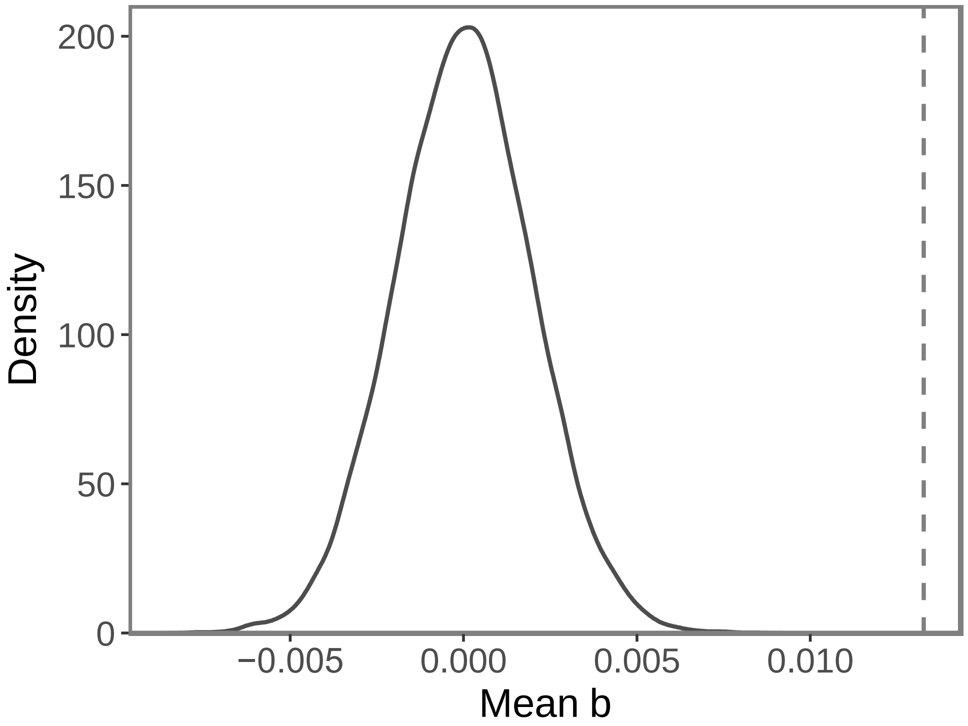

Supplementary Table S1. Kendall's τ correlation between the effect sizes of ADHD-associated SNPs with other psychiatric disorders considering the variants from three ancient datasets.

Psychiatric disorders		Ancient datasets (number of variants)		
		**Pre-Neolithic (3276 SNPs)**	**Near East (2707 SNPs)**	**Neolithic (3320 SNPs)**
**ASD**	τ	0.498	0.501	0.494
	P-value	<1.61e-315	1.61e-315	<1.61e-315
	SNPs of the ancient ADHD dataset included in the ASD GWAS	3275	2707	3320
**BD**	τ	0.110	0.107	0.106
	P-value	3.50e-20	6.83e-16	7.44e-19
	SNPs of the ancient ADHD dataset included in the BD GWAS	3264	2697	3307
**MDD**	τ	0.256	0.245	0.249
	P-value	5.20e-100	1.29e-75	1.01e-95
	SNPs of the ancient ADHD dataset included in the MDD GWAS	3241	2675	3281
**SCZ**	τ	0.086	0.075	0.069
	P-value	6.21e-13	1.39e-08	6.09e-09
	SNPs of the ancient ADHD dataset included in the SCZ GWAS	3276	2707	3320

ASD: Autism Spectrum Disorder, BD: Bipolar Disorder, MDD: Major Depression Disorder, SCZ: Schizophrenia

Supplementary Table S2. Online resources.

Deposited data (Ref)	Source
**Pre-Neolithic genotypes**[1]	*https://reich.hms.harvard.edu/datasets*
**Near East genotypes**[2]	
**Neolithic genptypes**[3]	
**Ancient Africans dataset**[4]	
**Altai Neanderthal Genome**[5]	*http://cdna.eva.mpg.de/neandertal/altai/AltaiNeandertal/VCF/*
**Modern Genomes (1000 Genomes Project Phase 3)**[6]	*ftp://ftp.1000genomes.ebi.ac.uk/vol1/ftp/release/20130502/*
**ADHD European GWAS**[7]	*https://ipsych.au.dk/downloads/*
**ASD**[8]**, BP**[9]**, MDD**[10] **and SCZ**[11] **GWAS**	*https://www.med.unc.edu/pgc/download-results/*
**SDS UK10K**[12]	*http://datadryad.org/resource/doi:10.5061/dryad.kd58f*
**Neanderthal-introgressed tagSNPs in modern humans**[13]	*http://akeylab.princeton.edu/downloads.html*

Supplementary Table S3. Prior distributions of the ABC_DL analysis. Node notation from Fig. S6.5 from *(26)*. T_X = time of node X. *U(A,B)* = uniform distribution between A and B. *N(x,y)* = normal distribution with mean x and standard deviation y.

Parameter	Prior distribution
**γ***	*U(-1e-6,1e-6)*
**genetic drift****	*U(0, 0.0000005)*
**ADHD_GS_root**	*N(-0.003,0.001)*
**T_split_Africa*****	*U(46021, 126000)*
**T_X*****	*U(T_split_Africa-7549, T_split_Africa)*
**T_western_Eurasian*****	*U(T_X -37471,T_X)*
**T_nc1e0*****	*U(T_western_Eurasian-37471, T_western_Eurasian)*
**T_ncle2*****	*U(T_ncle0-30011, T_ncle0)*
**T_nclc1*****	*U(T_ncle2-34796, T_ncle2)*
**T_ndle0*****	*U(T_nclc1-34796, T_nc1c1)*
**T_n1e0*****	*U(T_ndle0-18721, T_ndle0)*
**T_ndle2*****	*U(T_nle0-18721, T_nle0)*
**T_nf1f1*****	*U(T_nc1c1-30011, T_nc1c1)*
**T_n1e1*****	*U(T_nf1f1-30011, T_nf1f1)*
**T_n1e3*****	*U(nle1-8051, nle1)*
**T_nd1d1*****	*U(nle1-30011, nle1)*
**T_ndle3*****	*U(nd1d1-18721, nd1d1)*
**T_ncle3*****	*U(nd1d1-30011, nd1d1)*
**T_nle2*****	*U(T_nle0-8050,T_nle0);*
**T_nle4*****	*U(min(T_nle2,T_nle3)-8050, min(T_nle2,T_nle3))*
**T_ncle4*****	*U(min(T_ncle2,T_ncle3)-30011,min(T_ncle2,T_ncle3))*
**T_ndle4*****	*U(min(T_ndle2,T_ndle3)-18721,min(T_ndle2,T_ndle3))*

* by generation and SNP. Generation time = 29 years[14]

** by generation. Generation time = 29 years

*** years

References

1. Fu, Q. *et al.* The genetic history of Ice Age Europe. *Nature* **534**, 200–205 (2016).
2. Lazaridis, I. *et al.* Genomic insights into the origin of farming in the ancient Near East. *Nature* **536**, 419–24 (2016).
3. Lipson, M. *et al.* Parallel palaeogenomic transects reveal complex genetic history of early European farmers. *Nature* **551**, 368–372 (2017).
4. Skoglund, P. *et al.* Reconstructing Prehistoric African Population Structure. *Cell* **171**, 59-71.e21 (2017).
5. Prüfer, K. *et al.* The complete genome sequence of a Neanderthal from the Altai Mountains. *Nature* **505**, 43–49 (2014).
6. 1000 Genomes Project Consortium *et al.* A global reference for human genetic variation. *Nature* **526**, 68–74 (2015).
7. Demontis, D. *et al.* Discovery of the first genome-wide significant risk loci for attention deficit/hyperactivity disorder. *Nat. Genet.* **51**, 63–75 (2019).
8. Grove, J. *et al.* Identification of common genetic risk variants for autism spectrum disorder. *Nat. Genet.* **51**, 431–444 (2019).
9. Stahl, E. A. *et al.* Genome-wide association study identifies 30 loci associated with bipolar disorder. *Nat. Genet.* **51**, 793–803 (2019).
10. Howard, D. M. *et al.* Genome-wide association study of depression phenotypes in UK Biobank identifies variants in excitatory synaptic pathways. *Nat. Commun.* **9**, 1470 (2018).
11. Lam, M. *et al.* Comparative genetic architectures of schizophrenia in East Asian and European populations. *Nat. Genet.* **51**, 1670–1678 (2019).
12. Field, Y. *et al.* Detection of human adaptation during the past 2000 years. *Science (80).* **354**, 760–764 (2016).
13. Vernot, B. *et al.* Excavating Neandertal and Denisovan DNA from the genomes of Melanesian individuals. *Science (80).* **352**, 235–9 (2016).
14. Fenner, J. N. Cross-cultural estimation of the human generation interval for use in genetics-based population divergence studies. *Am. J. Phys. Anthropol.* **128**, 415–423 (2005).
